# Inoculation of *Bacillus velezensis* SD24 enhancing the accumulation of tea catechin secondary metabolites

**DOI:** 10.1128/spectrum.03469-25

**Published:** 2026-05-18

**Authors:** Liangliang Yu, Hongbo Li, Huilin Yu, Yu Zhou, Xu Wang, Li Luo

**Affiliations:** 1 Shanghai Key Laboratory of Bio-energy Crops, School of Life Sciences, Shanghai University 34747, Shanghai, China; 2 National Key Laboratory of Tea Plant Germplasm Innovation and Resource Utilization, College of Tea Science, Anhui Agricultural University 12486, Hefei, China; Pennsylvania State University, University Park, Pennsylvania, USA

**Keywords:** catechins, *Bacillus velezensis*, rhizosphere microbiome, transcriptional regulation, microbial antagonism

## Abstract

Tea (*Camellia sinensis*) is a globally significant economic crop, and its desirable quality and health benefits are largely credited to catechin derivatives. Plant growth-promoting rhizobacteria (PGPR), such as *Bacillus velezensis*, are well-known for enhancing the environmental fitness and disease resistance of plants. However, the regulation of their impact on tea catechin biosynthesis remains unclear. While previous studies have focused on PGPR-facilitated growth promotion in crops like tomatoes and rice, the physiological mechanisms by which microbes regulate secondary metabolism in tea—especially under co-inoculation conditions—remain largely underexplored. This study examined the effects of *B. velezensis* SD24, isolated from tea rhizosphere soil, on catechin derivative accumulation of tea leaves by altering gene expression and the rhizosphere microbiome. Strain SD24 exhibited broad-spectrum antimicrobial activity against various pathogens due to behaving antimicrobial gene clusters. Tea plants inoculated with SD24 showed significantly increased levels of catechin derivatives in their leaves. This was likely achieved by upregulation of leucoanthocyanidin reductase and anthocyanidin reductase within the phenylpropanoid pathway. Additionally, chlorophyll content was increased. Transcriptomic analysis revealed a notable enrichment in biosynthesis of secondary natural products among the tea genes activated by SD24 inoculation. Metagenomic analysis further demonstrated that SD24 inoculation led to a restructuring of the tea rhizosphere microbiome. Notably, co-inoculation with *Piriformospora indica*, a beneficial endophytic fungus, suppressed SD24-induced gene expression and catechin accumulation, underscoring its antagonism toward SD24. These findings suggest that *B. velezensis* SD24 enhances tea quality, probably by transcriptionally activating the synthesis of catechin derivatives, a process associated with the restructuring of the rhizosphere microbiome.

**IMPORTANCE:**

The mechanisms through which plant growth-promoting rhizobacteria (PGPR) influence secondary metabolism in perennial crops remain poorly understood. This study demonstrates that *Bacillus velezensis* SD24, a tea rhizosphere isolate, significantly enhances the accumulation of health-beneficial catechin derivatives in tea leaves. This quality improvement is associated with transcriptionally upregulating key biosynthetic genes (*LAR* and *ANR*) and concurrently restructuring the rhizosphere microbiome. Furthermore, we reveal a critical antagonistic interaction, where the beneficial fungus *Piriformospora indica* suppresses these SD24-induced effects. Our findings provide crucial insights into how specific PGPR strains may directly enhance tea quality by affecting host plant metabolism and the root microbiome, highlighting the complex and tailored microbial interactions that could be harnessed for sustainable agriculture.

## INTRODUCTION

Tea (*Camellia sinensis*) is the second most consumed beverage in the world after water, with over 50 countries and regions growing tea globally. Rich in natural bioactive substances, tea is the basis of its health benefits. Tea polyphenols are the most important functional components in tea, accounting for 18%–36% of the dry weight (1). Catechins make up 70%–80% of tea polyphenols, with epigallocatechin-3-gallate (EGCG) being the most active and abundant, known for its strong antioxidant capabilities, protection of cardiovascular health, antimicrobial and antiviral properties, anti-inflammatory effects, regulation of glucose metabolism, and neuroprotection (2). Other components like theanine, caffeine, and tea polysaccharides also have beneficial effects on human health.

Catechin biosynthesis starts with phenylalanine, which goes through the phenylpropanoid pathway to form coumaroyl-CoA; this is then combined with malonyl-CoA by chalcone synthase to form chalcone (3). Chalcone is converted into flavanone by chalcone isomerase, and flavanone is transformed into dihydroflavonol by flavanone 3-hydroxylase. Dihydroflavonol is reduced to leucoanthocyanidin by dihydroflavonol 4-reductase. Leucoanthocyanidin serves as a branching point, leading to the formation of catechins through two pathways (4). One pathway involves leucoanthocyanidin reductase (LAR) catalyzing the direct conversion of leucoanthocyanidin into catechins, while the other pathway involves leucoanthocyanidin being transformed into anthocyanidin by anthocyanidin synthase, followed by the conversion into epicatechin by anthocyanidin reductase (ANR). The synthesis of ester catechins, such as EGCG, starts with gallic acid, which is converted into βG by uridine diphosphate glucose [UDPG]-galloylglucose transferase; βG then acts as an activated donor, transferring galloyl groups to epicatechin in the presence of epicatechin gallate transferase (3). The synthesis of methylated catechins (e.g., EGCG3"Me) in tea plants is catalyzed by specific O-methyltransferases (CsFAOMT1 and CsFAOMT2), which methylate EGCG at the 3" or 4" positions, enhancing its stability and bioavailability (5). The expression of LAR and ANR is regulated by multiple transcription factors, including the MBW complex formed by MYB, bHLH, and WD40 proteins (6). In tea plants, the phosphate starvation response transcription factors CsPHR1 and CsPHR2 can activate ANR expression, while the jasmonate signaling pathway inhibitor CsJAZ3 interacts with CsPHR1/CsPHR2, integrating nutritional and hormonal signals to regulate catechin synthesis (7). There is little reported on whether the expression of catechin biosynthesis genes in tea is affected by the inoculation of plant growth-promoting rhizobacteria (PGPR).

PGPR plays a significant role in enhancing tea yield, improving quality, and promoting growth. When combined with selenium fertilizer, the application of *Bacillus amyloliquefaciens* increased the selenium content in the roots, leaves, and entire plant of tea trees (7, 8). The application of *Burkholderia phytofirmans* DFP-24 increased the total phosphorus content in tea leaves, elevated the level of free amino acids, reduced the phenol-ammonia ratio, and improved tea quality (9). The combined application of four rhizosphere-promoting bacteria, including *Pseudomonas putida*, with compound fertilizer resulted in a 12% increase in the weight of 100 buds, an 8.8% increase in germination density, an 18.3% increase in tea yield, and a 7.3% increase in amino acid content compared to the application of compound fertilizer alone (10).

*Bacillus velezensis*, a common rhizosphere-promoting bacterium, exhibits various growth-promoting mechanisms, including nutrient activation (e.g., phosphorus solubilization), secretion of plant hormones (e.g., IAA), and production of siderophores (11). It also produces a range of antimicrobial substances like fengycin and bacillomycin, as well as secretes enzymes such as cellulase and pectinase. *B. velezensis* can be used to control tea diseases such as bacterial blight and anthracnose, and it can also reduce the fluoride content in tea leaves (12). Additionally, it is effective against sugarcane smut, grape gray mold, peanut southern blight, barley diseases, and pests, and significantly promotes crop growth (13). In the rhizosphere of *Camellia oleifera*, *B. velezensis* CSUFT-BV4 effectively antagonizes *Colletotrichum* species by enhancing the activity of defense enzymes (superoxide dismutase, phenylalanine ammonialyase, and polyphenol oxidase) (14). Clearly, inoculating with *B. velezensis* can both increase tea yield and improve its quality. However, the regulatory mechanisms by which it enhances tea quality are not well understood.

This study investigates the effects of inoculating *B. velezensis* SD24 and *Piriformospora indica* (an endophytic fungus promoting plant tolerance to stress as a control) (15) on the levels of catechin compounds in tea, and reveals the possible physiological mechanisms from the transcriptional regulation and microbiome reconstruction.

## MATERIALS AND METHODS

### Microbial suppression assays of SD24

Antimicrobial activity of strain SD24 was evaluated against bacterial and fungal pathogens. For antibacterial assays, cultures of *Pseudomonas syringae pv. tomato* DC3000, *Pectobacterium carotovorum subsp. carotovorum* KC20, and *Xanthomonas campestris pv. campestris* 8004 were harvested from yeast extract broth (YEB) agar, suspended in 0.85% saline (OD_600_ = 0.8), and mixed into 50 mL of molten YEB medium before solidification. Wells (2 mm diameter) were created in the agar and filled with 5 µL of SD24 suspension (OD_600_ = 0.8). After incubation at 28°C for 48 h, inhibition zones were measured (16). Antifungal activity against *Piriformospora indica* was assessed on PDA plates by inoculating fungal spores centrally (17) and placing SD24 suspensions in peripheral wells (2 mm, spaced 1 cm apart). Plates were sealed and incubated at 28°C for 5 days before observation.

### 16S rDNA sequencing and phylogenetic analysis of SD24

Strain SD24 was isolated from the rhizosphere soil of tea plants collected from an ecological tea garden in Shucheng, Lu’an City, Anhui Province, China (31°28′46.1″N, 117°3′37.3″E). The isolate was cultured on YEB agar medium. Genomic DNA was extracted using a boiling method, where bacterial cells were heated in sterile water for 10 min. The 16S rDNA gene was amplified by PCR with universal primers 27F/1492R and PrimeSTAR Max DNA Polymerase (Takara) (18), under the following conditions: 30 cycles of denaturation at 94°C for 30 s, annealing at 56°C for 30 s, and extension at 72°C for 90 s. Amplified products were confirmed by 1% agarose gel electrophoresis and subsequently sequenced by Sangon Biotech (Shanghai). Based on BLAST analysis against the NCBI database, the strain showed >99% sequence similarity to closely related species. Phylogenetic analysis was performed using MEGA7 software to construct a Neighbor-Joining tree (19).

### Whole-genome sequencing and functional annotation of SD24

The whole genome of strain SD24 was sequenced by Benagen Technology (Wuhan, China). Genomic DNA was extracted from 1.0 g of bacterial cells using an optimized SDS-based method. DNA quality was assessed using a NanoDrop One spectrophotometer and Qubit 3.0 Fluorometer. Sequencing libraries were prepared with the SQK-LSK110 and EXP-NBD104/114 kits (Oxford Nanopore Technologies) for long-read sequencing on a PromethION platform, and the VAHTS Universal Plus DNA Library Prep Kit (Vazyme) for short-read sequencing on an Illumina NovaSeq 6000. After quality control, high-quality reads were *de novo* assembled and polished. Genome annotation included identification of repetitive sequences, coding genes, non-coding RNAs, genomic islands, and CRISPR arrays. Functional assignments were performed using Pfam, RefSeq, Nr, COG, KEGG, GO, and specialized databases such as CAZy, CARD, and VFDB. Detailed methods are available in the Benagen online manual (https://www.benagen.com/index.php?c=show&id=21).

### Tea plant cultivation and microbial inoculation

Tea seedlings (*Camellia sinensis* “Shuchazao,” stem diameter 3–5 mm) were cultivated in a 1:1 (vol/vol) mixture of natural soil and vermiculite in 30 cm diameter pots, with four plants per pot. *B. velezensis* SD24 was grown in YEB medium at 28°C with shaking at 150 rpm for 48 h, harvested by centrifugation, and resuspended in 0.85% NaCl. *P. indica* was cultured in seed liquid medium under the same conditions for 5–7 days, and spores were harvested and adjusted to OD_600_ = 0.02. For co-inoculation, equal volumes of SD24 and *P. indica* suspensions were mixed. Each treatment—SD24, *P. indica*, and their mixture—was applied to 20 uniformly sized seedlings (five pots) by drenching 1 mL of suspension around the root zone of each plant. Plants were maintained under controlled conditions: 16/8 h light/dark, 25°C, and 30%–40% humidity, with weekly irrigation. After 2 months, tender leaves, mature leaves, and rhizosphere soil were sampled for transcriptomic, metabolomic, and microbiome analyses.

### HPLC determination of natural products in tea

Catechin derivatives in tea leaves were quantified by high-performance liquid chromatography (HPLC) using a Waters 2695 system equipped with a UV/Vis detector (20). Briefly, 0.1 g of leaf powder was mixed with 5 mL of 70% methanol, and extracted for 20 min using an ultrasonic processor at room temperature. After centrifugation at 2,191 × *g* for 10 min, the extraction of the residue was repeated, and the combined supernatant was diluted to 10 mL, filtered through a 0.22 μm nylon membrane, and injected into the HPLC system. Separation was achieved on a Kinetex XB-C18 column (100 × 4.6  mm, 2.6 μm) with a 30°C column temperature, with 0.2% acetic acid (mobile phase A) and methanol (mobile phase B) as mobile phases. Catechin derivatives were detected and identified by comparing the retention times and UV spectra of standard solutions. Gallocatechin (GC), epigallocatechin (EGC), catechin (C), epicatechin (EC), caffeine (KAF), CG (catechin-3-gallate), epicatechin-3-gallate (ECG), EGCG, and gallocatechin-3-gallate (GCG) were identified by comparison with commercial standards (Sigma-Aldrich) and quantified using external calibration curves (20).

### Determination of chlorophyll in tea leaves

Chlorophyll content was determined from fresh, fully expanded tea leaves (21, 22) After surface rinsing with distilled water and gentle blot-drying, leaf samples were weighed and immersed in 8 mL of 95% ethanol in 15 mL centrifuge tubes. Samples were kept in darkness at room temperature with occasional agitation until complete decolorization was achieved. The extract was then brought to a final volume of 10 mL with 95% ethanol. Absorbance of 2 mL aliquots was measured at 665 nm and 649 nm using a UV spectrophotometer, with 95% ethanol as a blank. Chlorophyll a, chlorophyll b, and total chlorophyll concentrations (mg/g fresh weight) were calculated according to the following equations:

Chl a = (13.95 × A665 – 6.88 × A649) × *V*/(1,000 × Fw)

Chl b = (24.96 × A649 – 7.32 × A665) × *V*/(1,000 × Fw)

Total Chl = Chl a + Chl b

where *V* is the final volume (mL), and Fw is the fresh weight (g).

### Transcriptomic profiling of tea leaves

RNA extraction and transcriptome analysis were performed as follows. Total RNA was isolated from 1 g of fresh tea leaves using the JZol total RNA extraction kit (Majorbio, Shanghai). RNA quality was assessed using an Agilent 5300 Bioanalyzer and a NanoDrop ND-2000 spectrophotometer. Only samples meeting the following criteria were used for library construction: OD260/280 = 1.8–2.2, OD260/230 ≥ 2.0, RQN ≥ 6.5, 28S:18S ratio ≥1.0, and total RNA > 1 µg. Stranded mRNA libraries were prepared with the Illumina Stranded mRNA Prep, Ligation Kit, followed by size selection (300–400 bp) and PCR amplification. Sequencing was conducted on the NovaSeq X Plus platform (PE150). Raw reads were processed with fastp, and clean reads were aligned to the reference genome using HISAT2. Transcript assembly and quantification were performed with StringTie and RSEM, respectively. Differential expression analysis was carried out using DESeq2/DEGseq, with |log₂FC| ≥ 1 and false discovery rate (FDR) < 0.05 (DESeq2) or FDR < 0.001 (DEGseq) set as significance thresholds. Functional enrichment analysis (GO and KEGG) was performed using Goatools and scipy, with terms considered significant at a Bonferroni-corrected *P*-value < 0.05. Detailed protocols are available in the Majorbio online manual (https://www.majorbio.com/).

### qRT-PCR assay of key differential expression genes

Gene expression levels were validated by quantitative real-time PCR (qRT-PCR) (23). Total RNA was extracted from fresh tea leaves using the RNAprep Pure Plant Kit (Tiangen, Beijing). cDNA was synthesized with the PrimeScript RT Reagent Kit with gDNA Eraser (Takara, Dalian). qPCR amplification was performed in triplicate on a QuantStudio 6 Flex system using SYBR Advantage qPCR Premix (Takara), with the tea actin gene as an internal control. Gene-specific primers are listed in Table S1. Relative expression was calculated via the 2^–ΔΔCT^ method.

### Rhizosphere microbiome analysis

Microbiome analysis was performed on 1 g of tea rhizosphere soil. Sequencing was conducted by Benagen Technology (Wuhan, China) using Oxford Nanopore Technologies (ONT) long-read amplicon sequencing. The workflow included DNA extraction, target amplification, library preparation, and ONT sequencing. Raw data were processed and analyzed through quality filtering, taxonomic assignment, and diversity analysis within QIIME2. Alpha and beta diversity indices were calculated using the QIIME2 diversity module, with statistical comparisons performed by *t*-test or analysis of variance (ANOVA). Multiple visualizations were generated programmatically: species composition bar plots and ternary diagrams using Python matplotlib; clustering heatmaps with R pheatmap; phylogenetic trees constructed via QIIME2’s align-to-tree-mafft-fasttree pipeline and visualized with ggtree in R; and diversity curves (rarefaction, Shannon, rank abundance, and species accumulation) as well as ordination plots (principal component analysis [PCA], principal coordinates analysis [PCoA], Non-metric multidimensional scaling [NMDS], and partial least Squares discriminant analysis [PLS-DA]) using ggplot2. UPGMA trees and ANOSIM results were plotted with Python matplotlib. Detailed methods are available in the Benagen online manual (https://www.benagen.com/index.php?c=show&id=21).

### Statistical analysis

Experimental samples were collected from twenty tea plants. All quantitative experiments included three technical replicates and were repeated in two to three biological replicates. The error bars showed the standard deviation. For the significance analysis of differences in compound-level data and transcriptomic differential gene expression data, the Student’s *t*-test was used; for the significance analysis of differences in metagenomic sequencing data, ANOVA was used. *P* < 0.05 indicates a significant difference (*); *P* < 0.01 indicates a highly significant difference (**).

## RESULTS

### *B. velezensis* strain SD24 exhibits antimicrobial activity against plant pathogens

**Fig 1:**
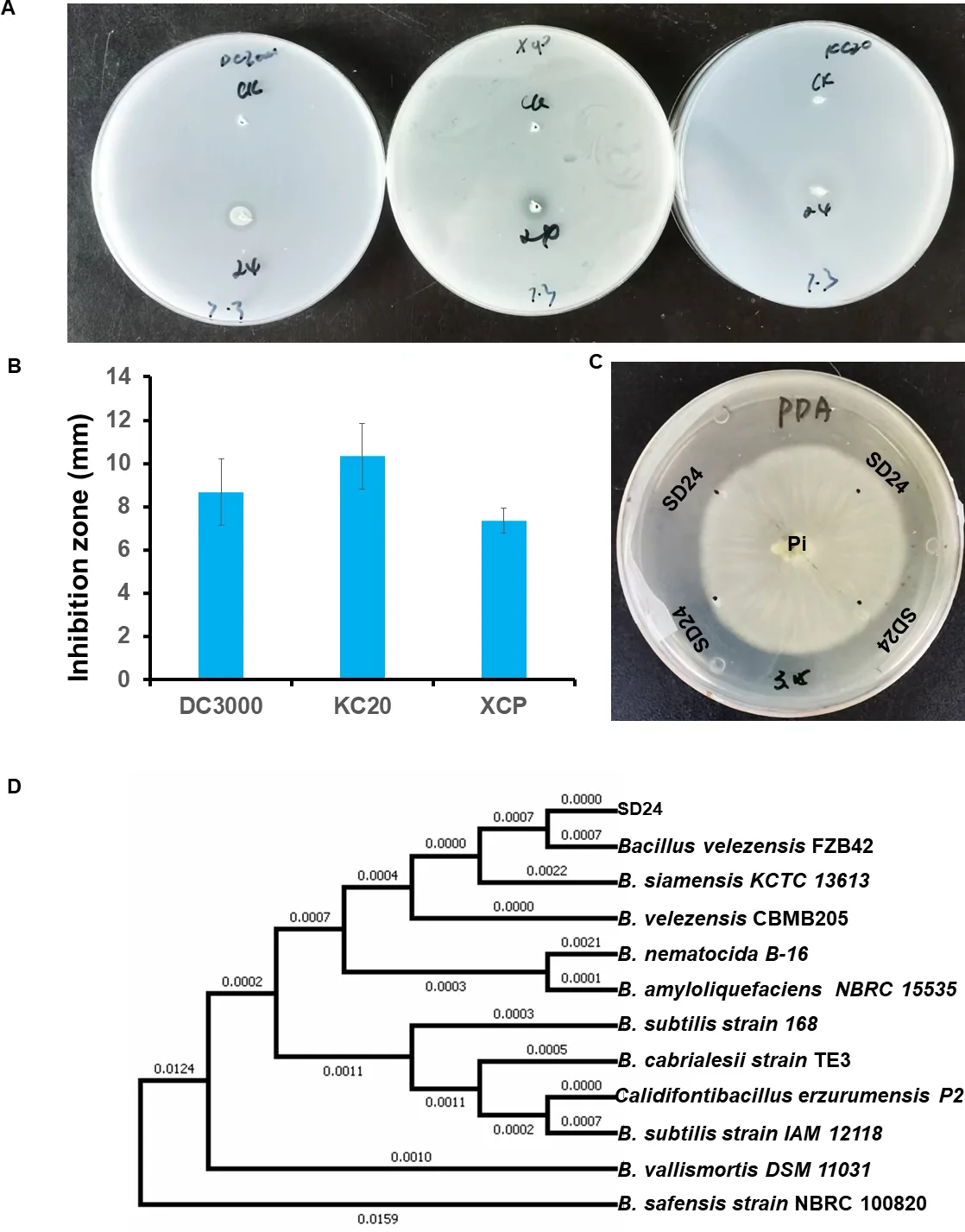
Identification and antimicrobial function of *Bacillus velezensis* SD24. (**A**) SD24 antagonized certain plant pathogenic bacteria. DC3000, *P. syringae* pv. *tomato* DC3000; XCP, *X. campestris pv. cmpestris* 8004; KC20, *P. carotovorum* subsp. c*arotovorum* KC20. (**B**) Inhibition zones of SD24 on the YEB agar plates containing plant pathogenic bacteria. (**C**) SD24 did not antagonize *P. indica*. Bars, 5 mm. (**D**) The position of SD24 on the 16s rDNA neighbor-joining molecular phylogenetic tree.

Bacterial co-culture experiments revealed that SD24, previously isolated from the rhizosphere soil of tea plants, effectively suppressed the growth of *P. syringae pv. tomato* DC3000, *X. campestris pv. cmpestris* 8004, and *P. carotovorum subsp. carotovorum* KC20 (Fig. 1A). The diameters of the inhibition zones reached 8.4 mm, 7.5 mm, and 10.5 mm, respectively (Fig. 1B, for KC20, the inhibition zone was not completely transparent). Additionally, we observed that SD24 did not show any significant suppressive effect against a plant endophytic fungus *P. indica* (Pi) (15) (Fig. 1C).

Comparative analysis of PCR-amplified 16s rDNA sequences indicated high homology between SD24 and *B. velezensis* FZB42 (coverage 95% and identity 98.84% [24]). Reconstructing the molecular phylogenetic tree confirmed that SD24 has the closest phylogenetic relationship with *B. velezensis* FZB42 (Fig. 1D). Therefore, it is named *B. velezensis* SD24. To evaluate the presence of antimicrobial gene clusters in the genome of SD24, its whole-genome DNA sequencing was completed, revealing a chromosome DNA sequence length of 3.91 M with a GC content of 46.66% (Fig. S1A), encoding 3,918 genes, including 3,721 CDS, 86 tRNA, 9 each of 23s rRNA, 16s rRNA, and 5s RNA, 1 tmRNA, 83 misc_RNA genes, and 202 repeat sequences. Analysis using antiSMASH software predicted that SD24 encodes the synthesis gene clusters for at least eight secondary metabolites, including surfactin, butirosin A/B, macrolactin H, bacillaene, fengycin, difficidin, bacillibactin, and bacilysin. Figure S1B illustrates the synthesis gene clusters of surfactin (*srfAA*, *srfAB*, *srfAC*, and *srfAD*), bacillaene (*pksJ1*, *pksL*, *pksM*, *pksN2*, and *pksR*), and difficidin (*pksJ2*, *fadA3*, *fabG4*, *acpP6*, and *pksE*). These encoding genes show high homology with those in FZB42 (amino acid sequence identity >99%).

### Impact of SD24 on tea plant development

**Fig 2:**
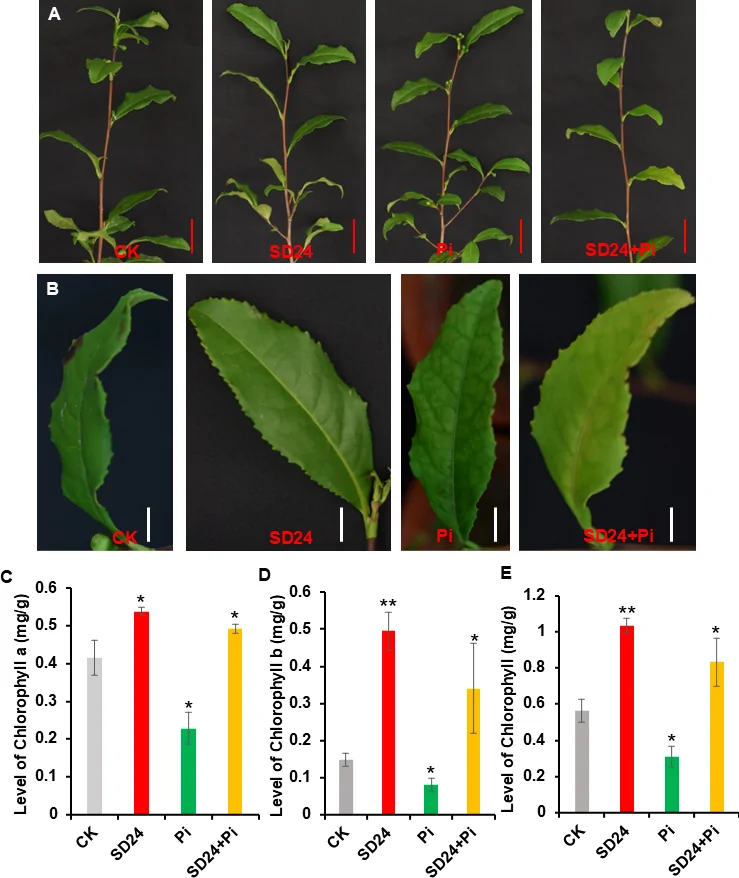
Growth and development of tea plants inoculated with *B. velezensis* SD24. (**A**) Tea plant size after 2-month inoculation of the microorganisms. (**B**) The first fully developed leaf from the top; Red bars, 10 cm; white bars, 1 cm. (**C–E**) Levels of chlorophyll in the tea leaves: CK, tea leaves without inoculation; SD24, tea leaves inoculated with *B. velezensis* SD24; Pi, tea leaves inoculated with *P. indica*; SD24 + Pi, tea leaves co-inoculated with *B. velezensis* SD24 and *P. indica*. Student’s *t*-test was used for statistical analysis, * indicates *P* < 0.05; ** indicates *P* < 0.01.

Considering that SD24 was isolated from the rhizosphere soil of tea plants, it is plausible that this strain could influence the growth, development, or metabolism of tea plants. To explore this, we cultivated tea plants in natural soil, using Pi as a control, either in single or mixed inoculations. After 2 months of inoculation, we observed no significant difference in plant height (approximately 27 cm, Fig. 2A). Interestingly, the leaves of tea plants inoculated with SD24 did not differ significantly from those that were not inoculated; in fact, they appeared slightly greener (Fig. 2B). However, some leaves of tea plants inoculated with Pi exhibited a pale-yellow color, and in tea plants co-inoculated with both microbes, some leaves turned yellow (Fig. 2B). Quantitative determination of chlorophyll levels in tea leaves showed that chlorophyll a levels were 29% and 18% higher in leaves inoculated with SD24 or co-inoculated with Pi, respectively, than in non-inoculated leaves respectively, while in leaves inoculated with Pi, it decreased by 45% (Fig. 2C). In leaves inoculated with SD24 or co-inoculated with Pi, the level of chlorophyll b increased by 2- and 1.3-fold respectively, whereas in leaves inoculated with Pi, it decreased by 46% (Fig. 2D). Overall, in leaves inoculated with SD24 or co-inoculated with Pi, the total chlorophyll level increased by 80% and 47%, respectively, while in leaves inoculated with Pi, it decreased by 45% (Fig. 2E). These results indicate that inoculation with SD24 significantly enhances chlorophyll levels in tea leaves, whereas inoculation with Pi has the opposite effect.

### Enhanced catechin accumulation in SD24-inoculated tea leaves

**Fig 3:**
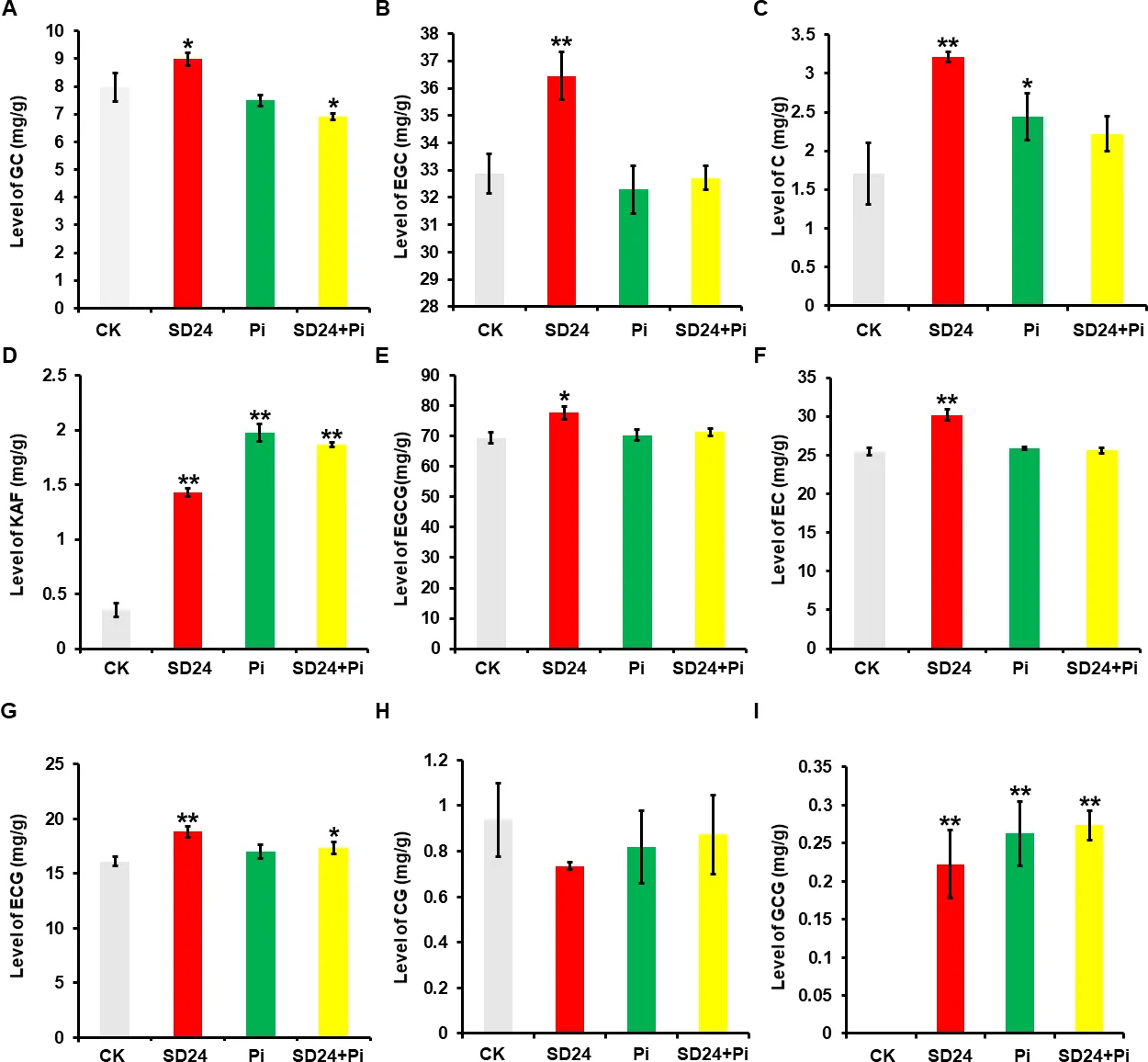
Effects of inoculation with *B. velezensis* SD24 on the levels of catechins and their derivatives in tea leaves (**A–I**). GC, gallocatechin; EGC, epigallocatechin; C, catechin; EC, epicatechin; KAF, caffeine; ECG, epicatechin-3-gallate; EGCG, epigallocatechin-3-gallate; CG, catechin-3-gallate; GCG, gallocatechin-3-gallate. *, *P* < 0.05; **, *P* < 0.01.

After a 2-month inoculation period with either SD24 or Pi, we collected tea leaves and analyzed the levels of secondary metabolites using HPLC. The results indicated that in tea leaves inoculated with D24, there was a significant increase in the levels of GC, EGC, C, EC, KAF, ECG, EGCG, and GCG (Fig. 3A through G and I; Fig. S2). Conversely, in tea leaves inoculated with Pi, only the levels of C, KAF, and GCG showed a significant increase (Fig. 3C and D; Fig. S2). In tea leaves co-inoculated with both microorganisms, only the levels of KAF, ECG, and GCG were significantly elevated, while the level of GC was notably decreased (Fig. 3A, D, G and I; Fig. S2). These findings suggest that inoculation with SD24 can significantly enhance the accumulation of catechin-related natural products, an effect that can be suppressed by concurrent inoculation with Pi.

### Transcriptomic changes in SD24-inoculated tea leaves

**Fig 4:**
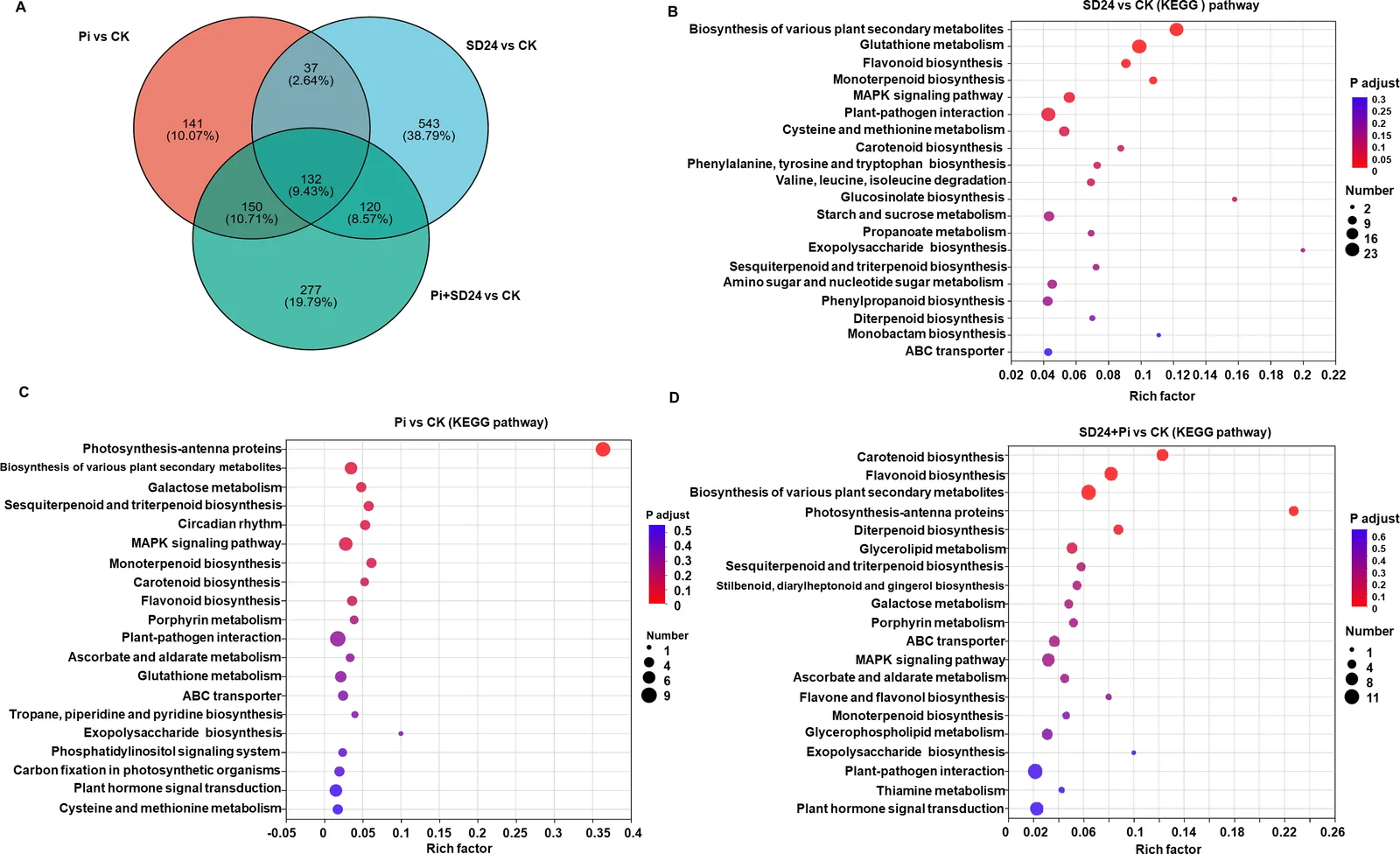
Transcriptome analysis of tea leaves inoculated with *B. velezensis* SD24. (**A**) Number of differentially expressed genes in tea leaves inoculated with *B. velezensis* SD24 or *P. indica*, compared to uninoculated tea leaves (Venn diagram). (**B–D**) KEGG enrichment of differentially expressed genes in tea leaves inoculated with *B. velezensis* SD24, *P. indica*, or co-inoculated with both microorganisms, compared to uninoculated tea leaves.

A transcriptome analysis of tea leaves, following a 2-month culture period with and without inoculation, revealed that 15,669, 16,239, 17,019, and 16,674 expressed genes were detected in the control group, tea leaves inoculated with SD24, Pi, and both microorganisms, respectively. Among these, 15,044 expressed genes were common to all four samples, while the number of genes uniquely expressed in each sample was 117, 197, 401, and 232, respectively. Further analysis of differentially expressed genes between the inoculated and control groups identified 832 genes differentially expressed in tea leaves inoculated with SD24 compared to the non-inoculated control (*Q* value > 0.5, *P* value < 0.01, with a fold change greater than two), 460 genes in Pi-inoculated tea leaves, and 679 genes in tea leaves inoculated with both microorganisms (Fig. 4A; Table S2). Among these, 132 genes were differentially expressed in all three comparisons, while the number of genes differentially expressed in only one comparison was 543 (SD24 vs CK), 141 (Pi vs CK), and 277 (SD24 + Pi vs CK), respectively (Fig. 4A). KEGG pathway enrichment analysis of the differentially expressed genes in each group revealed: (i) in tea leaves inoculated with SD24, the differentially expressed genes were significantly enriched in pathways related to plant secondary metabolism, flavonoid and monoterpenoid synthesis, glutathione metabolism, and the MAPK signaling pathway (Fig. 4B); (ii) in tea leaves inoculated with Pi, the differentially expressed genes were significantly enriched in pathways related to photosynthetic antenna proteins, synthesis of various plant secondary metabolites, galactose metabolism, sesquiterpenoid and triterpenoid synthesis, and the circadian rhythm pathway (Fig. 4C); (iii) in tea leaves inoculated with both microorganisms, the differentially expressed genes were significantly enriched in pathways related to carotenoid synthesis, flavonoid synthesis, various plant secondary metabolite synthesis, photosynthetic antenna proteins, and diterpenoid synthesis (Fig. 4D). These results indicate that inoculation with SD24 leads to the differential expression of multiple genes encoding enzymes involved in natural product synthesis in tea leaves.

### Upregulation of catechin biosynthesis genes by SD24

**Fig 5:**
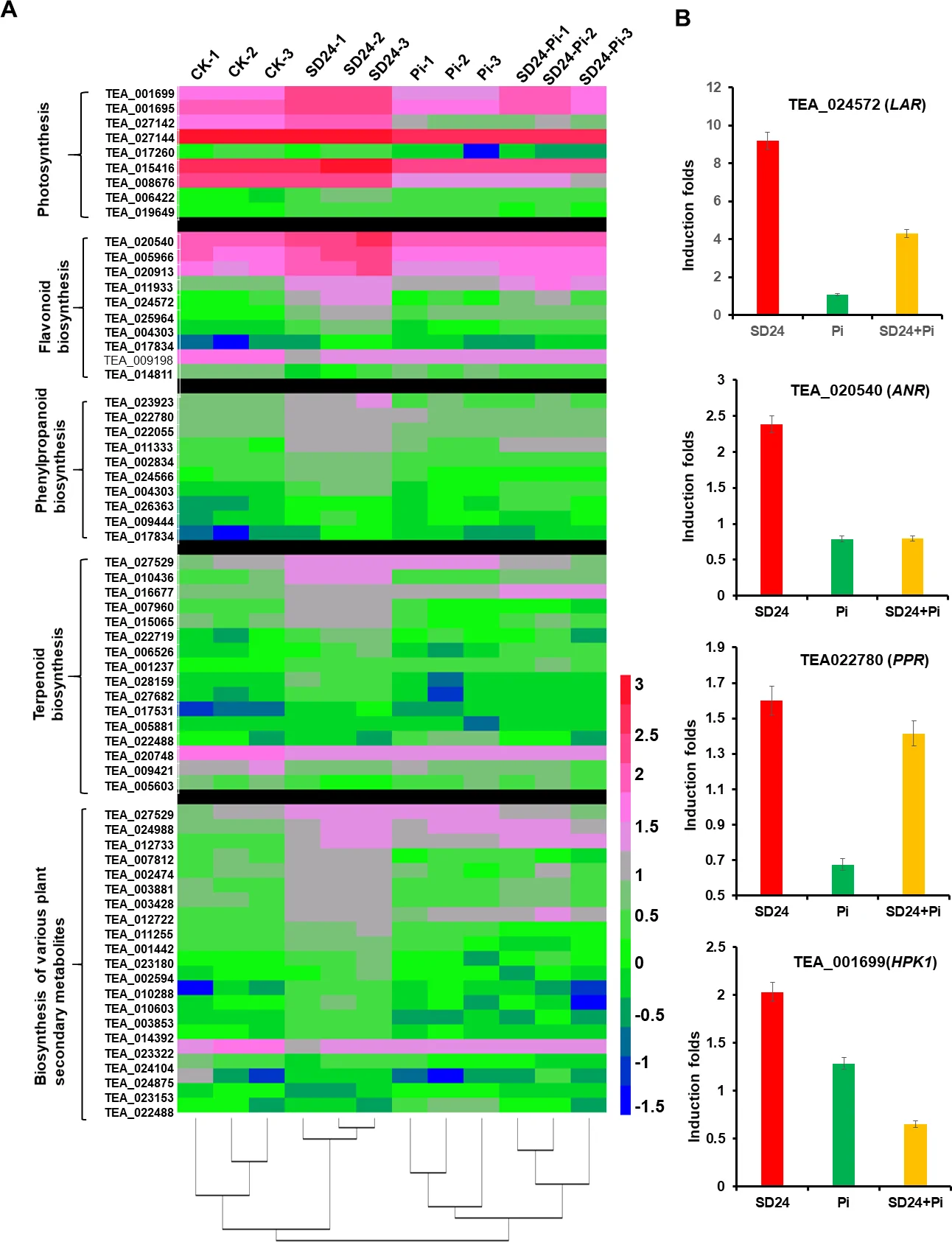
Inoculation with *B. velezensis* SD24 upregulated the expression of catechin synthase genes. (**A**) Transcriptomic difference heatmap of plant secondary metabolite genes in tea leaves inoculated with *B. velezensis* SD24 and co-inoculated with *B. velezensis* SD24 and *P. indica*. CK represents the transcripts of related genes in tea leaves without microbial inoculation. (**B**) Differential expression of catechin synthase genes *LAR*, *ANR*, *HPK1*, and *PPR* in tea leaves with and without microbial inoculation was analyzed by qRT-PCR. Induction folds: the level of mRNA from leaves of tea plants inoculated with the microorganism/the level of mRNA from leaves of tea plants without inoculation of microbes.

Further analysis of the differentially expressed secondary metabolite biosynthesis genes in tea plants inoculated with SD24, compared to uninoculated plants, categorized them into four groups: flavonoid biosynthesis (11 genes), phenylpropanoid biosynthesis (10 genes), terpenoid biosynthesis (16 genes), and other plant secondary metabolite synthesis (21 genes; Fig. 5A). Interestingly, only four of these differentially expressed genes (TEA_009198, TEA_015065, TEA_012722, and TEA_012733) showed differential expression in tea plants inoculated with Pi (Fig. 5A). This result suggests that SD24 induces a highly specific differential expression of secondary metabolite genes in tea plants. Surprisingly, in tea plants co-inoculated with both microorganisms, the corresponding differentially expressed genes enriched were reduced by more than half compared to those in tea plants inoculated with SD24 alone: six genes for flavonoid biosynthesis, four genes for phenylpropanoid biosynthesis, seven genes for terpenoid biosynthesis, and nine genes for other plant secondary metabolite synthesis (Fig. 5A). This result suggests that co-inoculation with Pi significantly suppresses the differential gene expression induced by SD24.

Careful analysis of the differentially expressed genes enriched in tea plants inoculated with SD24 revealed that the catechin biosynthesis genes *LAR* and *ANR* were induced 11- and 2-fold, respectively; however, these genes were not induced in tea plants inoculated with Pi (Table S2). In tea plants co-inoculated with both microorganisms, only *LAR* was induced threefold (Table S2).

To validate these transcriptome results, we applied qRT-PCR to analyze the expression levels of the *LAR*, *ANR*, and *PPR* (Pentatricopeptide repeat-containing protein) genes. The results showed that inoculation with SD24 significantly induced the expression of the *LAR* gene in tea leaves (induction ratio of 9.2-fold), whereas the induction effect was weakened upon co-inoculation with Pi (4.3-fold), and no significant induction was observed with Pi inoculation alone (Fig. 5B). Regarding the *ANR* gene, only inoculation with SD24 induced its expression (2.4-fold), while co-inoculation or inoculation with Pi alone did not induce expression (Fig. 5B). The *PRR* gene exhibited a similar induction expression trend to the LAR gene (Fig. 5B). These results suggest that inoculation with SD24 significantly induces the expression of key genes in the catechin biosynthesis pathway, while co-inoculation with Pi significantly suppresses this induction effect.

Furthermore, we also noted that after inoculation with SD24, the expression of some genes related to photosynthesis in tea leaves was induced (Fig. 5A). We used qRT-PCR to verify the expression of one of these genes (*HPK1*, Homeotic protein knotted-1, Fig. 5B), and the results obtained from the two methods were essentially consistent.

### Rhizosphere microbial community alterations by inoculation of SD24

**Fig 6:**
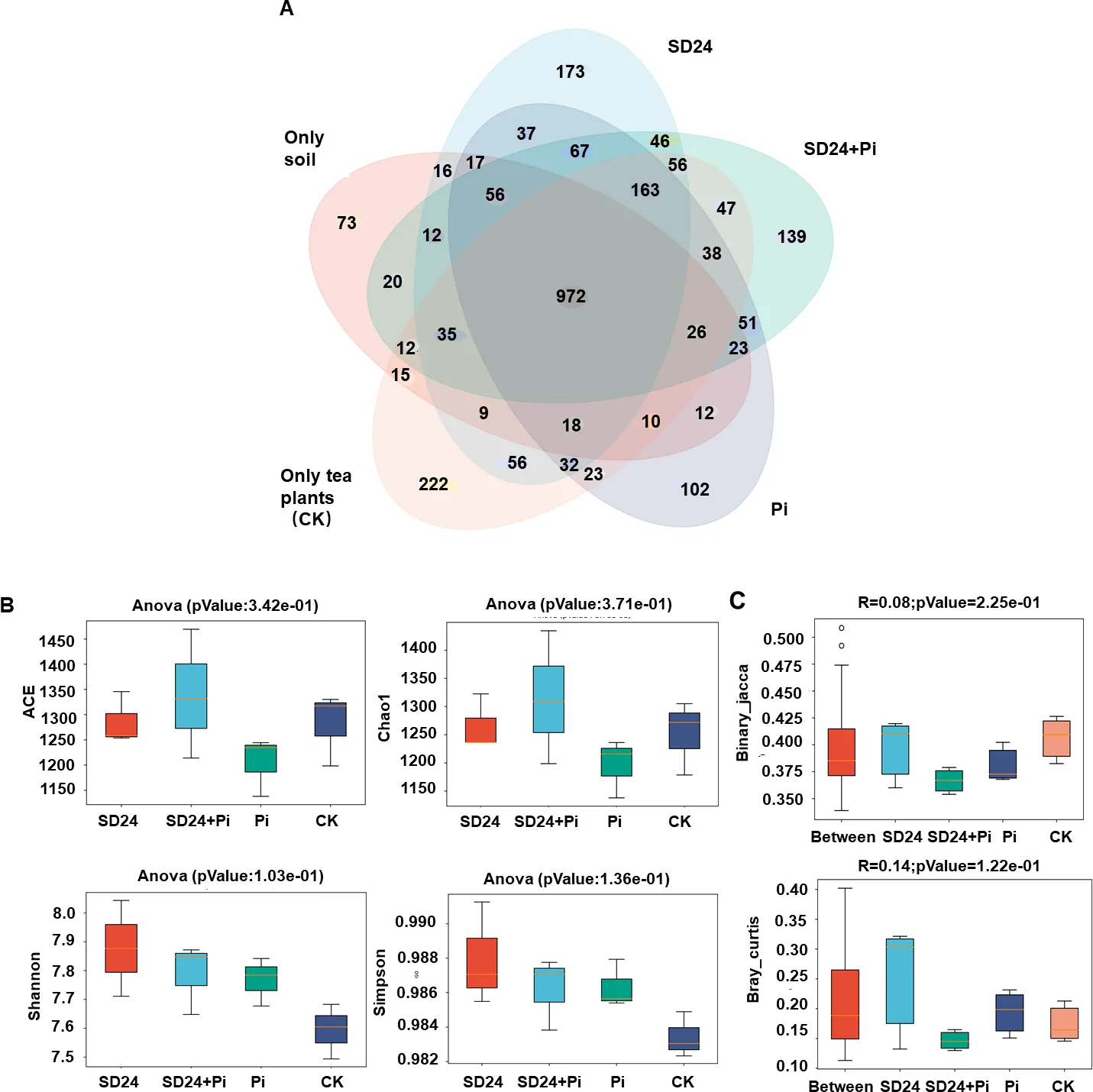
Diversity of the rhizosphere microbiome of tea plants inoculated with *B. velezensis* SD24. (**A**) The number of classified bacteria (OTU) in the rhizosphere of tea plants inoculated with *B. velezensis* SD24 or *P. indica*, respectively. (**B and C**) α- and β-diversity of the rhizosphere microbiome of tea plants.

**Fig 7:**
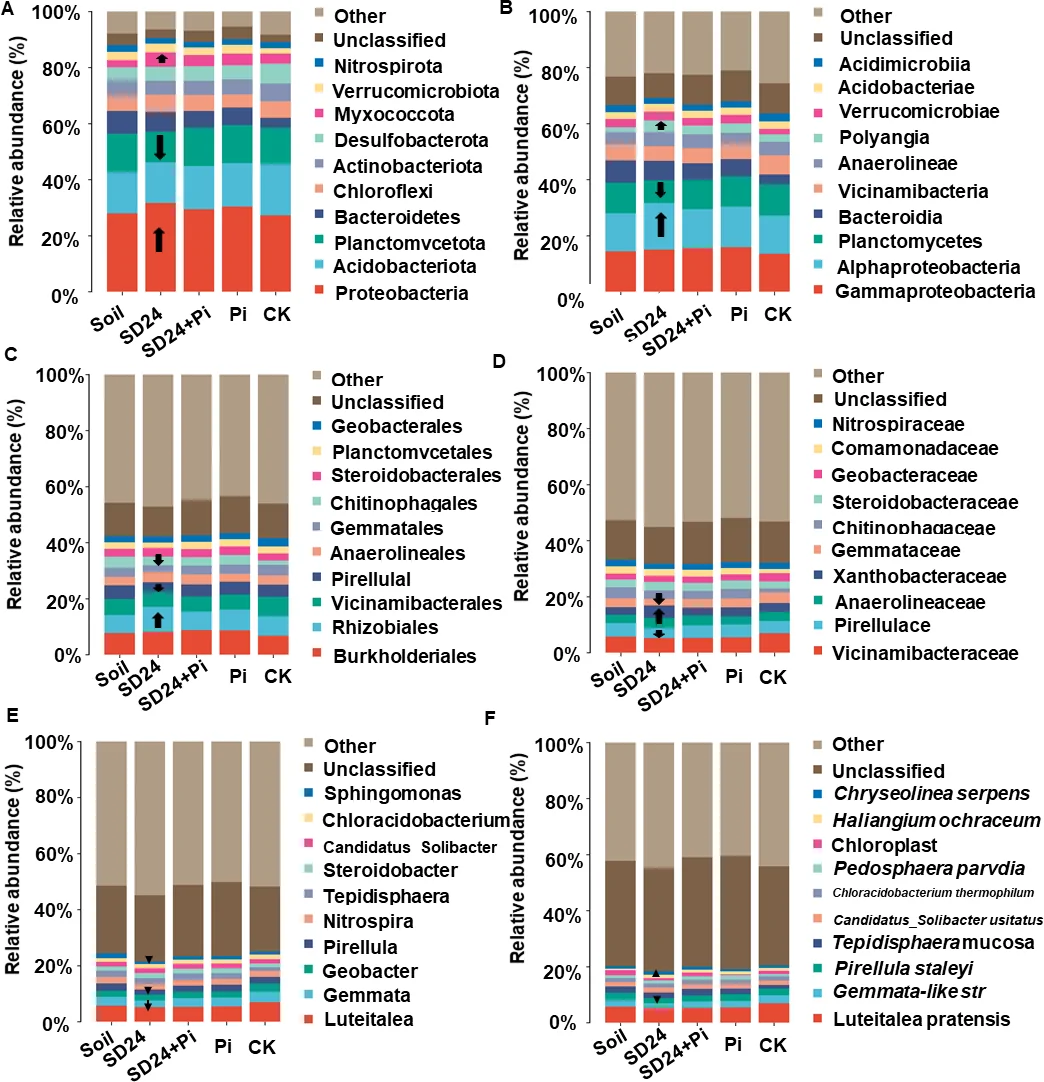
The relative abundance of dominant populations at different units. (**A–F**) Phylum, class, order, family, genus, and species. CK, rhizosphere soil of tea plants but without microbial inoculation; SD24, Pi, and SD24 + Pi rhizosphere soil of tea plants inoculated with *B. velezensis* SD24, *P. indica*, or both *B. velezensis* SD24 and *P. indica*. The black arrows represent an increase or decrease in abundance.

Inoculating SD24 in the rhizosphere of tea plants altered the accumulation of catechin secondary metabolites in tea leaves, possibly by changing the microbial community in the tea plant rhizosphere and subsequently influencing plant metabolism. To test this hypothesis, we employed metagenomic sequencing techniques to analyze the changes in the tea plant rhizosphere microbiota. The results indicated that 1,326, 1,734, 1,765, 1,647, and 1,763 OTUs were detected in natural soil without tea plants, in the rhizosphere soil of tea plants without microbial inoculation, inoculated with SD24, Pi, and both microorganisms, respectively (Fig. 6A). These findings suggest that planting tea plants significantly increases the variety of microorganisms in the soil, and inoculating SD24 may further enhance microbial diversity. α-Diversity analysis revealed that the ACE and Chao1 indices of the bacterial community in the rhizosphere soil of tea plants inoculated with SD24 were lower than that of co-inoculation or without inoculation, but higher than those inoculated with Pi (Fig. 6B). The Shannon index followed the order: SD24 > SD24 + Pi > Pi > no inoculation, and the Simpson index was similar to the Shannon index (Fig. 6B). These results suggest that the bacterial community in the rhizosphere soil of tea plants inoculated with SD24 was not particularly rich but evenly distributed, without forming dominant populations. β-Diversity analysis showed that species diversity followed the order: SD24 > no inoculation or Pi > SD24 + Pi (Fig. 6C), indicating that there were fewer shared bacterial species in the rhizosphere soil of tea plants inoculated with SD24 and the control strain Pi. Further analysis of the bacterial diversity in the rhizosphere soil of tea plants inoculated with different strains at various taxonomic levels (Phylum: Proteobacteria, Planctomycetota, and Myxococcota; Class: Alphaproteobacteria, Planctomycetes, and Polyangia; Order: Rhizobiales, Pirellulal, and Chitinophagales; Family: Pirellulaceae, Anaerolineaceae, Xanthobacteraceae, and Gemmataceae; Genus: Gemmata, Pirellula, and Sphingomonas; Species: Gemmata-like str and *Pirellula staleyi*) revealed a relatively even distribution of taxonomic groups, with changes in abundance observed in a few groups (Fig. 7A through D). These findings suggest that inoculating SD24 in the tea plant rhizosphere can alter the types and composition of soil microorganisms to some extent.

## DISCUSSION

The quality of tea is determined by the types and levels of catechin natural products, which are primarily synthesized via the phenylpropanoid pathway, a multi-enzyme catalytic route (3). Within this pathway, LAR and ANR are two key enzymes responsible for catechin and their derivative synthesis (4). Their expression is influenced by environmental signals such as low phosphorus and is regulated by transcription factors, including MYB and bHLH (6). Although PGPR is known to enhance tea plant growth and biotic stress tolerance (12), the mechanism by which PGPR inoculation affects tea quality—particularly catechin level—remains unclear. Here, we demonstrated that inoculation with *B. velezensis* SD24 strongly activated the expression of *LAR* and *ANR*, leading to catechin accumulation. In contrast, inoculation with the control strain Pi had no significant effect, and co-inoculation resulted in antagonistic outcomes. These changes were accompanied by the reconstruction of the rhizosphere microbiome. Our findings provide the first evidence that PGPR can elevate catechin content in tea through transcriptional regulation, offering insights for further mechanistic studies and supporting the potential use of PGPR in tea cultivation.

SD24, isolated from the tea rhizosphere soil, shares traits with other *B. velezensis* strains (e.g., CSUFT-BV4 and FZB42) (24–27), including strong antagonism against bacterial pathogens (Fig. 1A), likely due to its antimicrobial gene clusters (Fig. S1B). This suggests that other *B. velezensis* strains may possess similar functional potential. While SD24 upregulated *LAR* and *ANR* expression (Fig. 5) and increased catechin and chlorophyll levels (Fig. 2C through E and 3), it did not significantly promote tea plant growth (Fig. 2A), possibly due to plant variety and seedling age factors to be examined in future studies. The shift in rhizosphere microbiota after SD24 inoculation (Fig. 6 and 7) may arise from bacterial metabolites (Fig. S1B) or altered root metabolism. Transcriptional activation of *LAR* and *ANR* (Fig. 5) could be associated with changes in hormone levels and transcription factor activity, as some *B. velezensis* strains produce auxin (11) and activate the transcription factors MYB and bHLH to reprogram downstream gene expression (6); however, the specific hormones involved require further investigation.

Although Pi is generally regarded as a PGPR that improves abiotic stress tolerance in crops (15), its inoculation here did not enhance tea plant growth or chlorophyll content (Fig. 2), nor did it positively affect catechin levels (Fig. 3) or synthase gene expression (Fig. 5), indicating it may not be a beneficial microbe for tea. Notably, co-inoculation with Pi antagonized SD24-mediated increases in catechin accumulation and gene expression (Fig. 3 to 5), corresponding to changes in the rhizosphere microbiome composition (Fig. 6 and 7). The basis of this antagonism remains unclear, as SD24 did not inhibit Pi hyphal growth on PDA plates (Fig. 1C), suggesting that microbe–microbe interactions in the rhizosphere differ from those *in vitro*. This highlights the complexity of designing effective microbial consortia.

**Fig 8:**
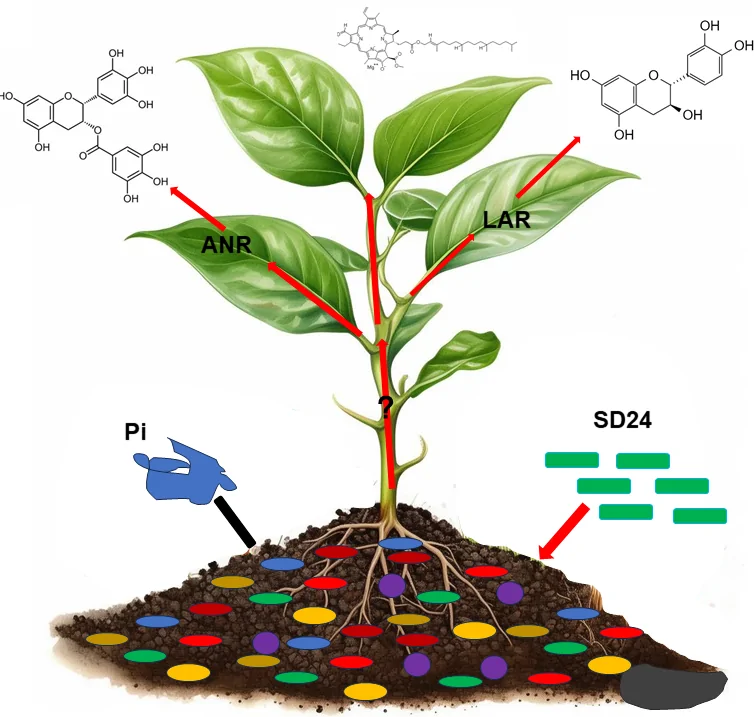
A possible model of *B. velezensis* SD24 affecting the accumulation of catechin compounds in tea leaves. The possible mechanism by which the inoculation of tea plants with *B. velezensis* SD24 increases the accumulation of catechin compounds and chlorophyll in tea leaves is as follows: It affects the rhizosphere microbiome, and then causes changes in the abundance of specific microbial groups and their metabolic products in the microbiome; SD24 itself and its products induce plant physiological changes, such as defense responses, hormone synthesis, and distribution.

In summary, SD24 inoculation activates catechin synthase genes LAR and ANR, raising catechin and chlorophyll levels and reshaping the rhizosphere microbiome—an effect suppressed by co-inoculation with Pi (Fig. 8). These results offer molecular insights into how PGPR influence plant secondary metabolism and provide a foundation for developing tailored microbial inoculants in tea agriculture.

## Data Availability

Original data of the whole genome of strain SD24, the transcriptome of tea leaves, and the metagenomes of the rhizosphere microbiome have been deposited in China National Center for Bioinformation (https://ngdc.cncb.ac.cn) under the accession numbers PRJCA056068, PRJCA056077, and PRJCA056080, respectively.

## SUPPLEMENTAL MATERIAL

Supplemental material spectrum.03469-25-s0001.docx Table S1; Fig. S1 and S2.

Table S2 spectrum.03469-25-s0002.xls Differential gene expression.

ASM does not own the copyrights to Supplemental Material that may be linked to, or accessed through, an article. The authors have granted ASM a non-exclusive, world-wide license to publish the Supplemental Material files. Please contact the corresponding author directly for reuse.
