# Supplementary material for: Inoculation of Bacillus velezensis SD24 enhancing the accumulation of tea catechin secondary metabolites
Source: Microbiol Spectr. 2026 May 18;14(7):e03469-25. doi: 10.1128/spectrum.03469-25 (PMC13339961; doi:10.1128/spectrum.03469-25)
Supplement: Supplemental material — Table S1; Fig. S1 and S2. [file spectrum.03469-25-s0001.docx]

**Supplemental materials**

**Table S1 Primers of qRT-PCR used in this study;**

**Tables S2 differential gene expression in tea leaves after inoculation of microbes**; **Figure S1 Genomic DNA sequencing of SD24**

1. The whole genome map of SD24. **B.** Three antibacterial gene clusters in the SD24 genome.

**Figure S2 Graphs of HPLC assays**

HPLC graphs of standard chemicals (**A**) and extracts from the tea leaf samples (**B**).

**Figure S1**

**
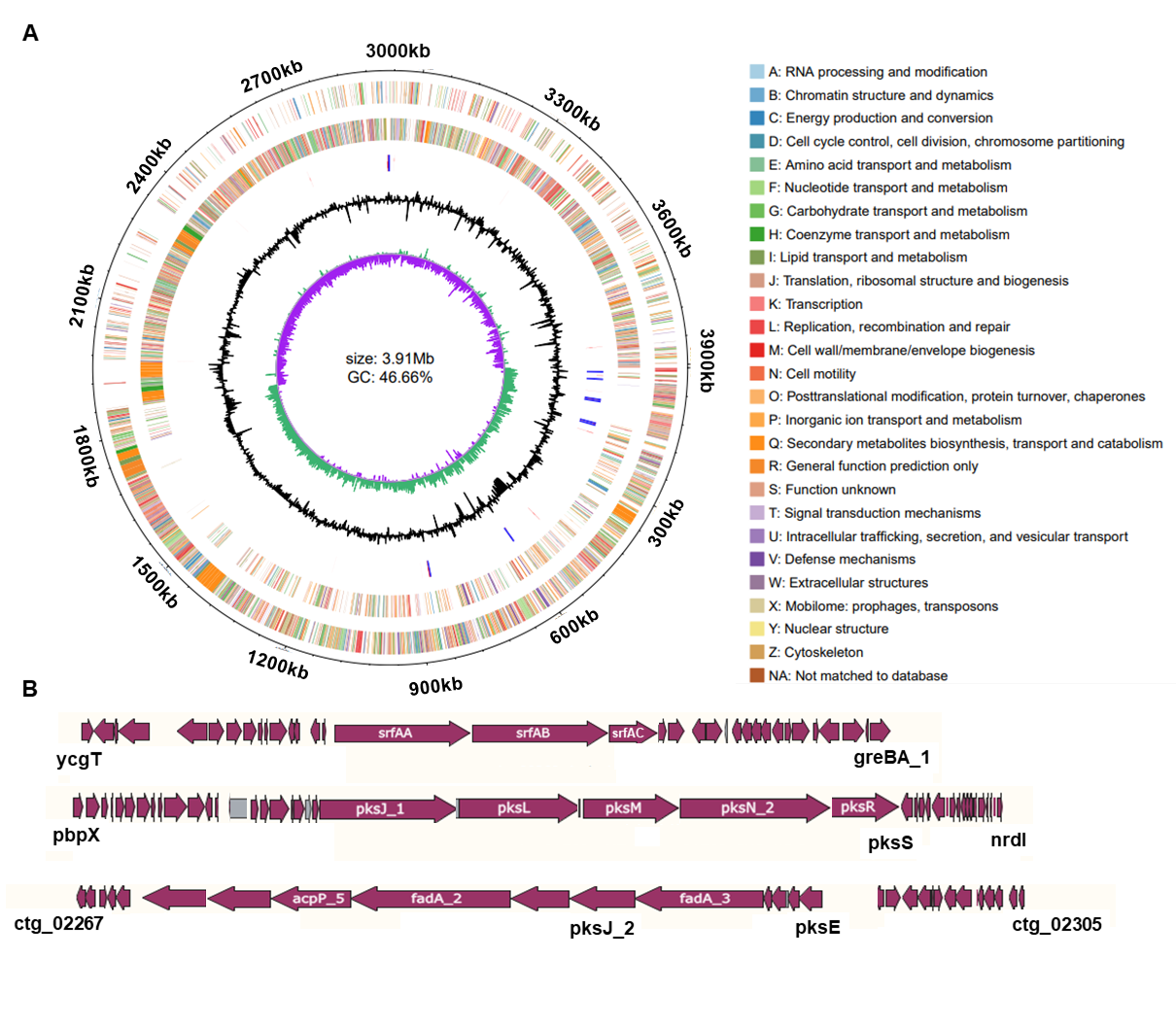
**

**Figure S2**

**
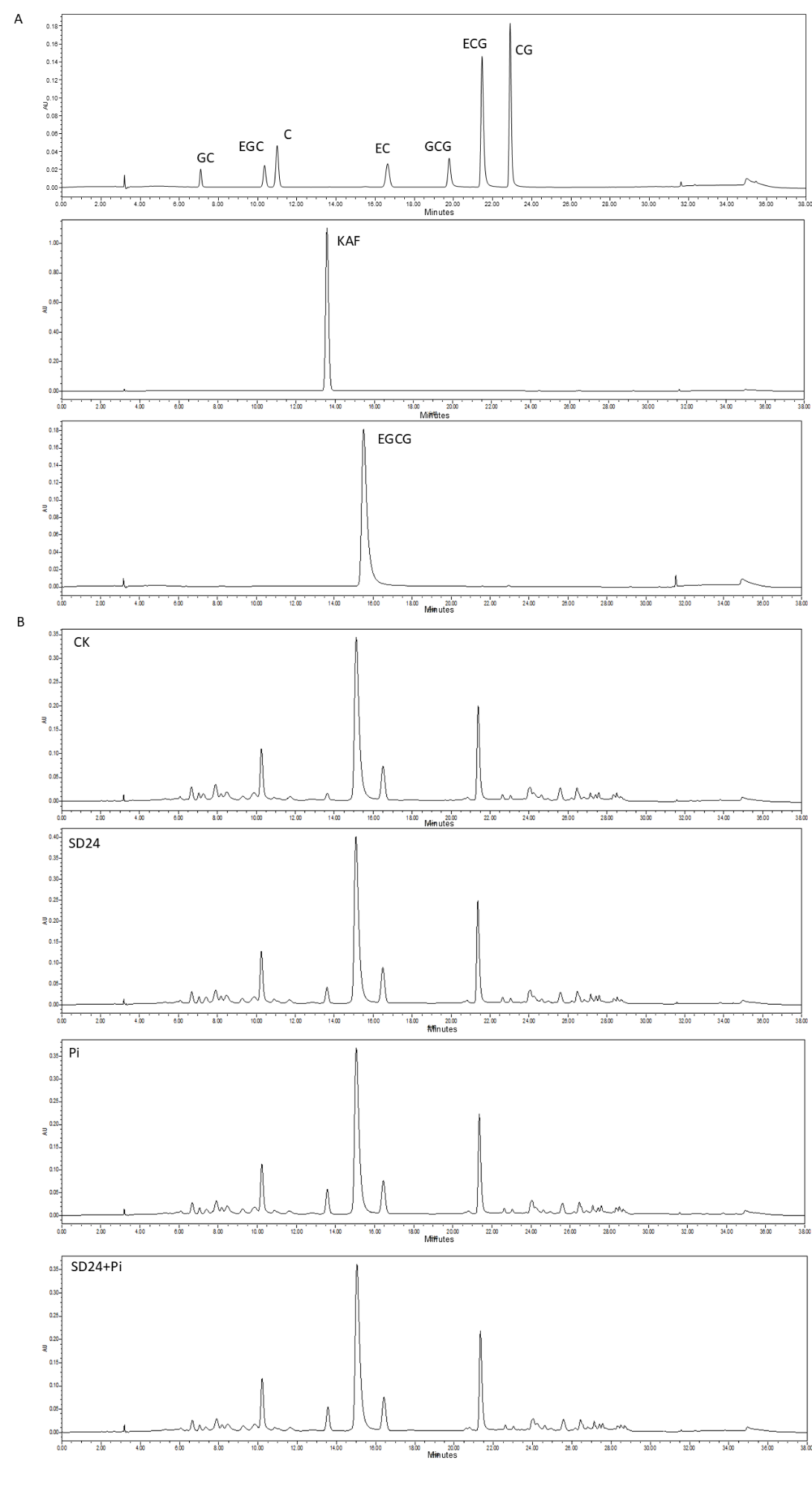
**

**Table S1 Primers of qRT-PCR used in this study**

| **Primer** | **Oligo (5'to3')** |
| --- | --- |
| LAR-qF | ACTAGACCAACTCACCCTAG |
| LAR-qR | ACTCTTCTATCAATCGCCGC |
| ANR-qF | AGCAGACCTCACCGATGAAC |
| ANR-qR | ACACGTTTAACCGTTCCTGC |
| 01699-qF | GTCAAGACTGAAGCCGAAAC |
| 01699-qR | GACAGTCCATGTAGGCTTCC |
| 22780-qF | GCGTGTAGAGGTACGGATTC |
| 22780-qR | CACCATGCCAATCTAATCTC |
|  |  |
| Tea Actin qF | TGACATGGAGAAGATCTGGC |
| Tea Actin qR | TGTATGGCGACATACATAGC |
